# Macrophage-Mediated Inflammatory Disorders

**DOI:** 10.1155/2013/316482

**Published:** 2013-06-13

**Authors:** I-Ming Jou, Chiou-Feng Lin, Kuen-Jer Tsai, Sung-Jen Wei

**Affiliations:** ^1^Department of Orthopedics, College of Medicine, National Cheng Kung University, Tainan 704, Taiwan; ^2^Institute of Clinical Medicine, College of Medicine, National Cheng Kung University, Tainan 704, Taiwan; ^3^Department of Pharmacology, The University of Texas Health Science Center, San Antonio, TX 78229-3900, USA

Mediators of inflammation induced by macrophages are critical for a variety of human inflammatory disorders, such as sepsis-related multiple organ dysfunction/multiple organ failure, microbial infection, acute brain/lung/hepatic/renal injuries, neurodegenerative disorders, tumorigenesis, osteoporosis/osteonecrosis, cardiovascular and metabolic diseases, and autoimmune diseases. Most of these cases are proinflammatory and pathogenic for disease progression, once activated macrophages actively secrete and cause an imbalance of cytokines, chemokines, and mediators of inflammation. The primary focus of this special issue is on the current and updated knowledge of macrophage-mediated inflammatory disorders, particularly on the pathogenesis of the macrophage activation syndrome, mediators of inflammation and anti-inflammation, and strategies against such effects. Lots of papers were submitted and reviewed by Editors and Reviewers, and twenty-four papers are accepted for publication. The brief introductions of these papers are as follows.

Due to the profile of released mediators (such as cytokines, chemokines, and growth factors), neoplastic cells modulate the activity of immune system, directly affecting its components both locally and peripherally. A. Eljaszewicz et al. reviewed, in the paper entitled *“Collaborating with the enemy: function of macrophages in the development of neoplastic disease,”* the specific functions of macrophages in the development of neoplastic disease.

The study called *“Regulatory role of GSK-3*β* on NF-*κ*B, nitric oxide, and TNF-*α* in group A streptococcal infection,”* investigates the interaction between GSK-3*β*, NF-*κ*B, and possible related inflammatory mediators in vitro and in a mouse model. The results revealed that GAS could activate NF-*κ*B, followed by an increased expression of inducible nitric oxide synthase (iNOS) and NO production in a murine macrophage cell line. Y.-T. Chang et al. demonstrated that the inhibition of GSK-3*β* to moderate the inflammatory effect might be an alternative therapeutic strategy against GAS infection.

During the early and short inflammatory phase, macrophages exert proinflammatory functions like antigen-presenting phagocytosis and the production of inflammatory cytokines and growth factors that facilitate the resolution of inflammation. However, persistence of proinflammatory activity and altered function of macrophages result in the development of chronic inflammatory diseases such as AD. In *“Role of macrophages in the pathogenesis of atopic dermatitis,”* S. Kasraie and T. Werfel highlight the new findings on dysregulated function of macrophages, the importance of these cells in the pathogenesis of AD in general, and the contribution of these cells in enhanced susceptibility against microbial infections in particular.

C. E. Boorsma et al. in *“Macrophage heterogeneity in respiratory diseases”* provided an overview of what macrophage phenotypes have been described, what their known functions are, what is known about their presence in the different obstructive and restrictive respiratory diseases (asthma, COPD, and pulmonary fibrosis), and how they are thought to contribute to the etiology and resolution of these diseases.

In asthma, an important role for innate immunity is increasingly being recognized. Key innate immune cells in the lungs are macrophages. In *“Characterization of macrophage phenotypes in three murine models of house-dust-mite-induced asthma”* C. Draijer et al. demonstrated the balance between macrophage phenotypes changes as the severity of allergic airway inflammation increases. Influencing this imbalanced relationship could be a novel approach to treat asthma.

Neuropathic syndromes which are evoked by lesions to the peripheral or central nervous system are extremely difficult to treat, and available drugs rarely joint an antihyperalgesic effect with a neurorestorative effect. N-Palmitoylethanolamine (PEA) exerts antinociceptive effects in several animal models and inhibits peripheral inflammation in rodents. In *“Palmitoylethanolamide is a disease-modifying agent in peripheral neuropathy: pain relief and neuroprotection share a PPAR-alpha-mediated mechanism”* these results from L. D. C. Mannelli et al. strongly suggest that PEA, via a PPAR-*α*-mediated mechanism, can directly intervene in the nervous tissue alterations responsible for pain, starting to prevent macrophage infiltration.

Several studies have provided evidence with regard to the neuroprotection benefit, so hyperbaric oxygen (HBO) therapy incases stroke, and HBO also promotes bone marrow stem cells (BMSCs) proliferation and mobilization. In the paper entitled *“Long course hyperbaric oxygen stimulates neurogenesis and attenuates inflammation after ischemic stroke”* Y.-S. Lee et al. find that mobilization of BMSCs to an ischemic area is more improved in long course HBO treatments, suggesting the duration of therapy is crucial for promoting the homing of BMSCs to ischemic brain by HBO therapies. HBO also can stimulate expression of trophic factors and improve neurogenesis and gliosis.

Indoleamine 2,3-dioxygenase 1 (IDO1), the L-tryptophan-degrading enzyme, plays a key role in the immunomodulatory effects on several types of immune cells. Originally known for its regulatory function during pregnancy and chronic inflammation in tumorigenesis, the activity of IDO1 seems to modify the inflammatory state of infectious diseases. Y. Murakami et al., in *“Remarkable role of indoleamine 2,3-dioxygenase and tryptophan metabolites in infectious diseases: potential role in macrophage-mediated inflammatory diseases,”* offer insights into new therapeutic strategies in the modulation of viral infection and several immune-related disorders.

Effective repair of damaged tissues and organs requires the coordinated action of several cell types, including infiltrating inflammatory cells and resident cells. In *“Macrophage plasticity and the role of inflammation in skeletal muscle repair,”* Y. Kharraz et al. summarize how ordered changes in macrophage polarization, from M1 to M2 subtypes, can differently affect muscle stem cell (satellite cell) functions. In addition, they highlight some of the new mechanisms underlying macrophage plasticity and briefly discuss the emerging implications of lymphocytes and other inflammatory cell types in normal versus pathological muscle repair.

As a facultative intracellular pathogen, *Staphylococcus aureus* invades macrophages and then promotes the cytoprotection of infected cells thus stabilizing safe niche for silent persistence. This process occurs through the upregulation of crucial antiapoptotic genes, in particular, myeloid cell leukemia-1 (Mcl-1). In *“The role of Mcl-1 in S. aureus-induced cytoprotection of infected macrophages,”* J. Koziel et al. report that *S. aureus* is hijacking the Mcl-1-dependent inhibition of apoptosis to prevent the elimination of infected host cells, thus allowing the intracellular persistence of the pathogen, its dissemination by infected macrophages, and the progression of staphylococci diseases.

The P2X7 purinergic receptor is a ligand-gated cation channel expressed on leukocytes including microglia. This study aimed to determine if P2X7 activation induces the uptake of organic cations, reactive oxygen species (ROS) formation, and death in the murine microglial EOC13 cell line. In *“P2X7 receptor activation induces reactive oxygen species formation and cell death in murine EOC13 microglia,”* R. Bartlett et al. demonstrate P2X7 activation induces the uptake of organic cations, ROS formation, and death in EOC13 microglia.

In *“Pivotal roles of monocytes/macrophages in stroke”* the reports from T. Chiba and K. Umegaki suggest that inflammation might directly affect the onset of stroke. Microglial cells and blood-derived monocytes/macrophages play important roles in inflammation in both onset and aggravation of stroke lesions. 

Macrophages play crucial roles in atherosclerotic immune responses. Recent investigation into macrophage autophagy in atherosclerosis has demonstrated a novel pathway through which these cells contribute to vascular inflammation. In *“Macrophage autophagy in atherosclerosis,”* M. C. Maiuri et al. discuss the role of macrophages and autophagy in atherosclerosis and the emerging evidence demonstrating the contribution of macrophage autophagy to vascular pathology. Finally, they show how autophagy could be targeted for therapeutic utility.

Dexamethasone (Dex) has been used to reduce inflammation in preterm infants with assistive ventilation and to prevent chronic lung diseases. In *“The role of glucocorticoid receptors in dexamethasone-induced apoptosis of neuroprogenitor cells in the hippocampus of rat Pups,”* C.-I. Sze et al. indicate early administration of Dex results in apoptosis of neural progenitor cells in the hippocampus, and this is mediated through glucocorticoid receptors.

Macrophages are innate immune cells derived from monocytes, which, in turn, arise from myeloid precursor cells in the bone marrow. Macrophages have many important roles in the innate and adaptive immune response, as well as in tissue homeostasis. In *“Alternatively activated macrophages in types 1 and 2 diabetes,”* A. Espinoza-Jiménez et al. review the advantages and disadvantages of two macrophage populations with regard to their roles in types 1 and 2 diabetes.

Macrophage migration inhibitory factor (MIF) is a proinflammatory cytokine, and the predictive role and pathogenic mechanism of MIF deregulation during kidney infections involving acute kidney injury (AKI) are not currently known. In *“Urinary macrophage migration inhibitory factor serves as a potential biomarker for acute kidney injury in patients with acute pyelonephritis,”* M.-Y. Hong et al. showed that elevated urinary MIF levels accompanied the development of AKI during kidney infection in patients with acute pyelonephritis (APN). An elevated urinary MIF level, along with elevated IL-1 *β* and KIM-1 levels, is speculated to be a potential biomarker for the presence of AKI in APN patients.

Peroxisome proliferator-activated receptors (PPARs) are shown to modulate the pathological status of sepsis by regulating the release of high mobility group box 1 (HMGB1), a well-known late proinflammatory mediator of sepsis. In *“Activation of peroxisome proliferator-activated receptor *γ* by rosiglitazone inhibits lipopolysaccharide-induced release of high mobility group box 1,”* J. S. Hwang et al. showed PPARs play an important role in the cellular response to inflammation by inhibiting HMGB1 release.

In the paper entitled *“Macrophages, inflammation, and tumor suppressors: ARF, a new player in the game,”* P. G. Través et al. provide an overview of the immunobiology of tumor-associated macrophages as well as what is known about tumor suppressors in the context of immune responses. Recent advances regarding the role of the tumor suppressor ARF as a regulator of inflammation and macrophage polarization are also reviewed.

Monocytes express many cell surface markers indicative of their inflammatory and activation status. Whether these markers are affected by diabetes and its complications is not known and was investigated in this study. In *“Alterations in monocyte CD16 in association with diabetes complications,”* D. Min et al. provide the evidence suggesting that the circulating monocyte phenotype is altered by diabetic complications status. These changes may be causally related to and could potentially be used to predict susceptibility to diabetic complications.

Inflammation is implicated in the development and rupture of atheromatous plaques, and there is considerable evidence supporting the involvement of adipocytokines in this inflammatory process. In *“Increased expression of visfatin in monocytes and macrophages in male acute myocardial infarction patients,”* C.-A. Chiu et al. provide another explanation about leukocytes mediated visfatin that may play a pathogenesis role in coronary vulnerable plaques rupture.

The lung is exposed to a vast array of inhaled antigens, particulate matter, and pollution. Cells present in the airways must therefore be maintained in a generally suppressive phenotype so that excessive responses to nonserious irritants do not occur; these result in bystander damage to lung architecture, influx of immune cells to the airways, and consequent impairment of gas exchange. In *“Macrophage-mediated inflammation and disease: a focus on the lung,”* E. G. Findlay and T. Hussell discuss the mechanisms behind this macrophage-mediated pathology, in the context of a number of inflammatory pulmonary disorders.

Most tissues harbor resident mononuclear phagocytes, that is, dendritic cells and macrophages. In *“Tissues use resident dendritic cells and macrophages to maintain homeostasis and to regain homeostasis upon tissue injury: the immunoregulatory role of changing tissue environments,”* M. Lech et al. report that organ- and disease phase-specific microenvironments determine macrophage and dendritic cell heterogeneity in a temporal and spatial manner, which assures their support to maintain and regain homeostasis in whatever condition. Mononuclear phagocytes contributions to tissue pathologies relate to their central roles in orchestrating all stages of host defense and wound healing, which often become maladaptive processes, especially in sterile and/or diffuse tissue injuries.

Different monocytic subsets are important in inflammation and tissue remodeling; although heart failure (HF) is associated with local and systemic inflammation, their roles in HF are yet unknown. In *“Changes in the monocytic subsets CD14* ^*dim*^ *CD16* ^+^ *and CD14* ^++^ *CD16* ^−^ *in chronic systolic heart failure patients,”* O. Amir et al. indicate the inverse association between EDD values and the expansion of CD14^dim^ CD16^+^ monocytes that can produce IL-13 which could be explained as a measure to counterbalance adverse remodeling, which is a central process in HF.

Traditional risk factors for metabolic disorders, including the waist circumstance, body mass index (BMI), triglyceride (TG), and ratio of TG to high density lipoprotein (HDL) cholesterol, were closely correlated with homoeostasis model assessment (HOMA) index in patients with nondiabetic RA. In *“Increased toll-like receptor 2 expression in peptidoglycan-treated blood monocytes is associated with insulin resistance in patients with nondiabetic rheumatoid arthritis”* S.-W. Wang et al. show the expressions of TLR2 in peripheral blood monocytes, following stimulation with peptidoglycan which is known as a TLR2 agonist, were closely correlated with the HOMA index, TNF-*α* and IL-6 concentrations. Accordingly, TLR-2 receptor and its related inflammatory cytokines could be potential therapeutic targets in managing insulin resistance in RA patients.

